# Machine learning’s effectiveness in evaluating movement in one-legged standing test for predicting high autistic trait

**DOI:** 10.3389/fpsyt.2024.1464285

**Published:** 2024-10-17

**Authors:** Yoshimasa Ohmoto, Kazunori Terada, Hitomi Shimizu, Hiroko Kawahara, Ryoichiro Iwanaga, Hirokazu Kumazaki

**Affiliations:** ^1^ Department of Behavior Informatics, Faculty of Informatics, Shizuoka University, Shizuoka, Japan; ^2^ Department of Electrical, Electronic, and Computer Engineering, Faculty of Engineering, Gifu University, Gifu, Japan; ^3^ Department of Neuropsychiatry, Graduate School of Biomedical Sciences, Nagasaki University, Nagasaki, Japan; ^4^ Unit of Medical Science, Nagasaki University Graduate School of Biomedical Sciences, Nagasaki, Japan

**Keywords:** autistic trait, machine learning, balance, one-legged standing, screening

## Abstract

**Introduction:**

Research supporting the presence of diverse motor impairments, including impaired balance coordination, in children with autism spectrum disorder (ASD) is increasing. The one-legged standing test (OLST) is a popular test of balance. Since machine learning is a powerful technique for learning predictive models from movement data, it can objectively evaluate the processes involved in OLST. This study assesses machine learning’s effectiveness in evaluating movement in OLST for predicting high autistic trait.

**Methods:**

In this study, 64 boys and 62 girls participated. The participants were instructed to stand on one leg on a pressure sensor while facing the experimenter. The data collected in the experiment were time-series data pertaining to pressure distribution on the sole of the foot and full-body images. A model to identify the participants belonging to High autistic trait group and Low autistic trait group was developed using a support vector machine (SVM) algorithm with 16 explanatory variables. Further, classification models were built for the conventional, proposed, and combined explanatory variable categories. The probabilities of High autistic trait group were calculated using the SVM model.

**Results:**

For proposed and combined variables, the accuracy, sensitivity, and specificity scores were 1.000. The variables shoulder, hip, and trunk are important since they explain the balance status of children with high autistic trait. Further, the total Social Responsiveness Scale score positively correlated with the probability of High autistic trait group in each category of explanatory variables.

**Discussion:**

Results indicate the effectiveness of evaluating movement in OLST by using movies and machine learning for predicting high autistic trait. In addition, they emphasize the significance of specifically focusing on shoulder and waist movements, which facilitate the efficient predicting high autistic trait. Finally, studies incorporating a broader range of balance cues are necessary to comprehensively determine the effectiveness of utilizing balance ability in predicting high autistic trait.

## 1 Introduction

Autism spectrum disorder (ASD) is a complex neurodevelopmental condition characterized by deficits in social communication and social interactions and repetitive, restricted behaviors (1). According to the United States Centers for Disease Control and Prevention, approximately 1 in 36 children is diagnosed with ASD (2). Further, the lifetime social cost associated with ASD is estimated to be approximately $3.6 million per individual (3). Research evidences that early intervention programs, particularly those initiated before the commencement of elementary education, can significantly enhance social functioning outcomes in children with ASD (4–7). Although ASD can be detected as early as 14 months of age (8) and diagnosed with high accuracy by 2 years of age (9), recent prevalence reports indicate that more than 70% of affected children are not diagnosed until after 51 months of age (10). Therefore, it is crucial to identify autistic traits in children by screening them prior to the initiation of elementary education.

Balance ability is the human body’s capacity to maintain postural stability in both static and dynamic states, and one must possess it to effectively perform daily activities (11, 12). The maintenance of balance requires the coordinated operation and integration of multiple mechanisms, including the sensory system, the central nervous system, and effectors (13). The sensory input system informs the body about its position relative to the surrounding environment. This sensory information is filtered, integrated, and processed by the central control system, which subsequently issues commands to effectors. A deficiency in balance ability not only increases the risk of falls among affected children which diminishes their motivation to participate in physical activities (14). Furthermore, balance impairments can hinder the development of communication and motor skills, limit the children’s integration into mainstream society, and adversely affect their social adaptation abilities (15).

Many studies indicate the presence of various motor impairments, including deficiencies in balance coordination, in children with ASD (16–19). Behavioral analyses clarify that children with ASD exhibit a significantly smaller center-of-pressure (COP) shift, a repetitive COP pattern, and less complex postural control compared to unaffected children. These impairments are evident in both static (18) and dynamic (19) balance states. Consequently, balance ability assessments of children with ASD are of paramount importance.

The one-legged standing test is a widely used assessment of balance (20–22). This test evaluates an individual’s ability to perform common everyday activities, such as walking, climbing stairs, and dressing; accordingly, the test is directly related to functional mobility and independence. Typically, measurements are taken twice for each leg, and the duration for which a subject can stand on one leg, along with maintaining postural sway and balance, is analyzed. The test can be administered in a short amount of time, thereby enabling the rapid screening of balance capabilities. Studies report that individuals with ASD exhibit significant static balance impairments while keeping their eyes open during the one-legged standing test (23, 24). However, there are limitations to the objective evaluation of these impairments.

Recent years have witnessed rapid advancements in machine learning technologies, with neural network technologies significantly affecting various domains, including video recognition. Since machine learning can effectively develop predictive models from movement data, the use of video recordings and machine learning algorithms to objectively evaluate movements during the one-legged standing test is feasible.

In this study, we conduct an experiment at a local preschool during the pupils’ medical examinations, which involve nearly all the 5-year-old residents (approximately 99% of the population of this age) of the selected city. Conducting research in this setting and focusing on a narrow age range (exclusively 5-year-old children) provided practical and relevant data. The objective of this study is to assess the effectiveness of using machine learning to evaluate movements during the one-legged standing test to predict high autistic trait group.

## 2 Materials and methods

### 2.1 Participants

This study was approved by the Ethics Committee of Nagasaki University, Japan (22051204). All participants were recruited from Sazacho, Nagasaki Prefecture, Japan. Nearly all the children residing in Sazacho participated in this study. All procedures involving human participants were conducted in accordance with the ethical standards of institutional and national research committees, as well as the 1964 Declaration of Helsinki and its subsequent amendments or comparable ethical standards. After receiving a comprehensive explanation of the study, all subjects and their guardians provided written informed consent to participate in the study. The participants satisfied the following inclusion criteria: All were 5-year-old residents of Sazacho.

**Figure 1:**
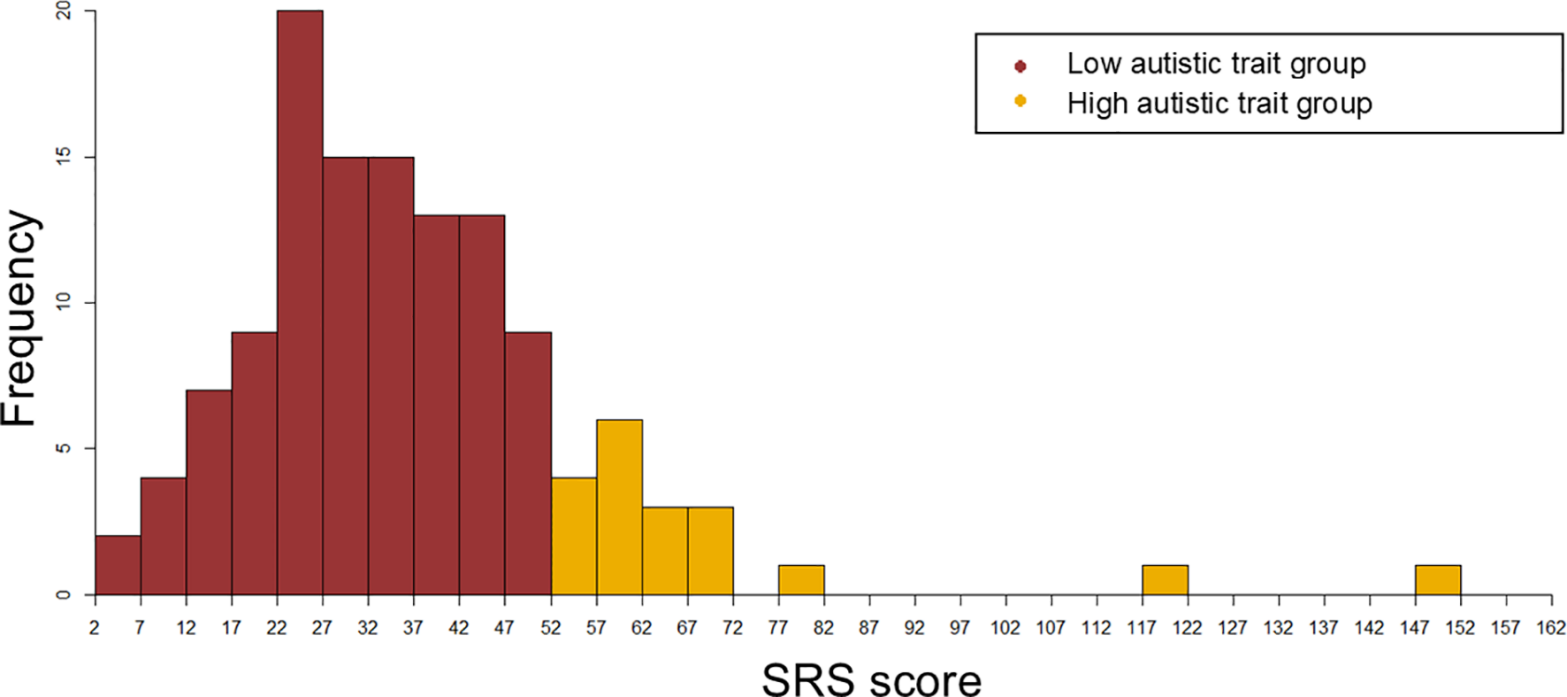
The distribution of the SRS scores in the High autistic trait group and Low autistic trait group.

To screen for clinically significant autistic symptoms, the participants’ mothers completed the Social Responsiveness Scale, Second Edition (SRS-2) (25). Higher the SRS score, higher the degree of autistic traits. Participants were classified into two groups, those having high autistic trait (High autistic trait group) and those having low autistic trait (Low autistic trait group), based on screening cutoff values (boys: 53.5, girls: 52.5), as described earlier reports on SRS score distribution in the Japanese population (26, 27). In this study, 64 boys and 62 girls participated. **Table 1** depicts the participants’ characteristics. **Figure 1** depicts the distribution of the SRS scores in the two groups.

**Table 1:** Participant characteristics.

	High autistic trait group (n=19) Mean (SD)	Low autistic trait group (n=107) Mean (SD)	Statistics t or χ^2^ df *p*
Age	5.13 (0.16)	5.06 (0.13)	1.717 22 0.07
Male/female ratio	9: 10	55: 52	0.746 1 0.81
SRS-2	69.79 (23.66)	31.24 (11.48)	-6.956 20 <0.001 **

SD, standard deviation, SRS-2, Social Responsiveness Scale, Second Edition.

**p < 0.001.

### 2.2 Apparatus

**Figure 2:**
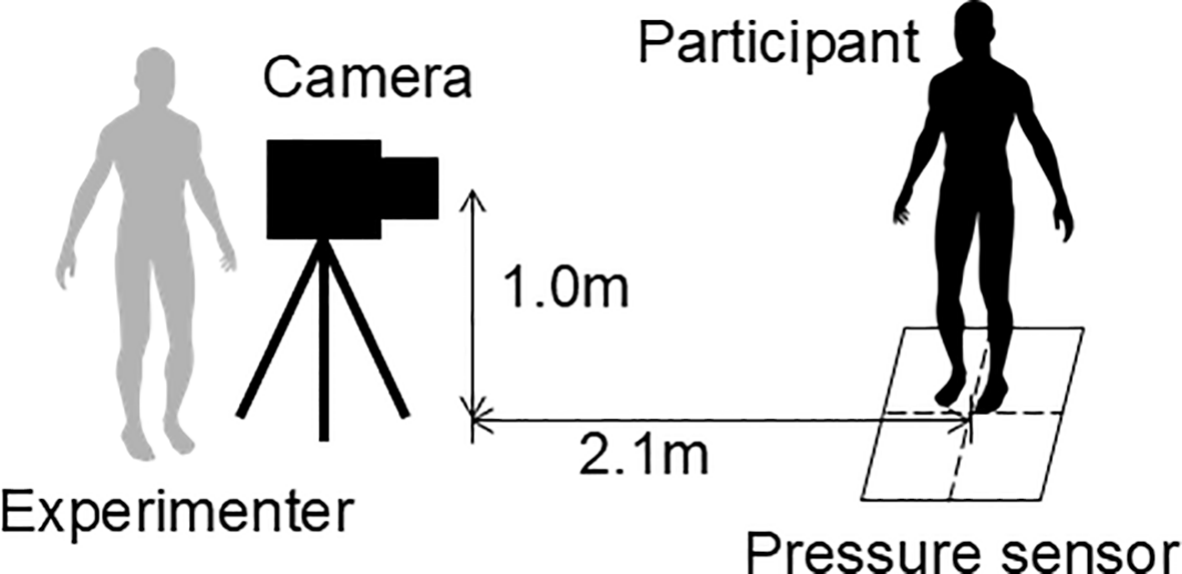
Spatial arrangement of the participant, experimenter, video camera, and pressure sensor during data acquisition.

A 48-cm square pressure sensor (LL sensor, NEWCOM, Inc.) was used to acquire data on the pressure distribution in the sole of the foot while participants were standing on one leg. To estimate the participants’ body motions, full-body images were captured using a webcam (Logitech c615n, 640 × 480 pixels, 30 fps) placed in front of them. Further, the data from the pressure sensor and the body images were synchronously acquired at a recording rate of 20 Hz. **Figure 2** depicts the configuration among the participant, experimenter, video camera, and pressure sensor during data acquisition.

### 2.3 Procedure

Participants stood on a pressure sensor while facing the experimenter, who directed them to start and stop standing on one leg. Each participant completed four one-legged standing tests, two on each leg, alternating between their right and left foot. Whenever a participant maintained the one-legged stance for more than 20 seconds, the trial ended. The trials were conducted consecutively. Each participant took 2 minutes or less to complete the entire experimental procedure.

### 2.4 Measurement

**Figure 3:**
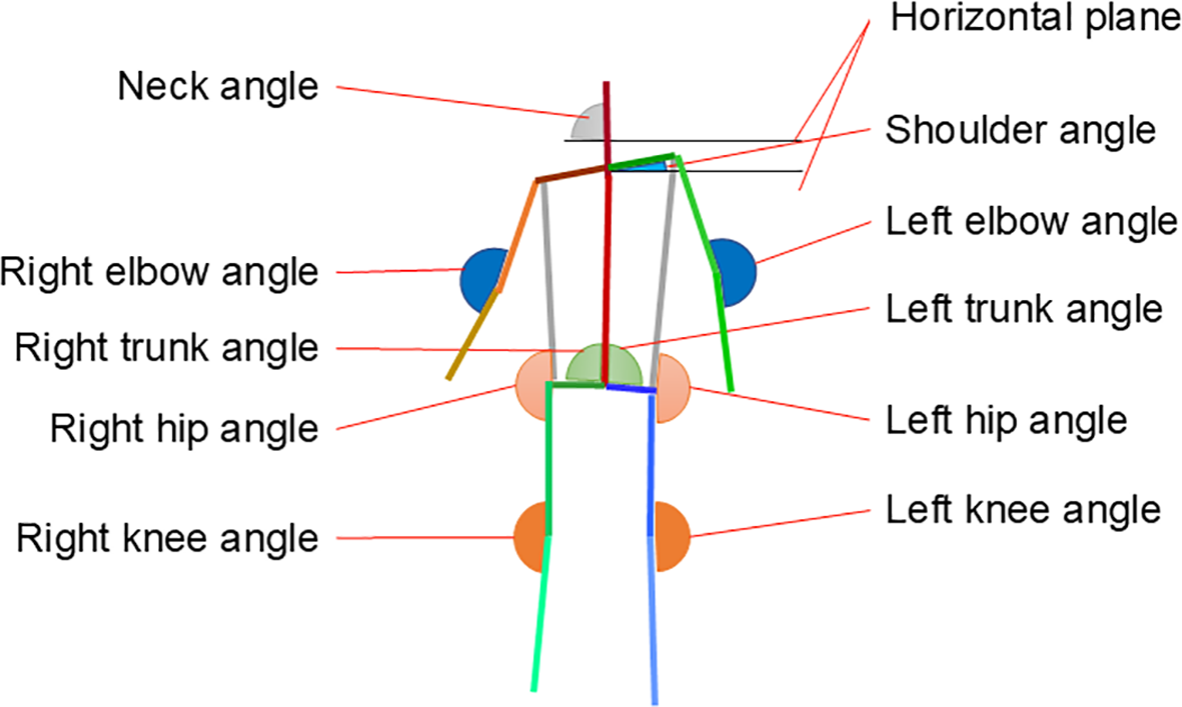
Estimated positions and names of various joints.

The data collected in the experiment were time-series data of the pressure distribution on the sole of the foot and full-body images of the participants standing on one leg. COP was estimated from the pressure distribution that was obtained when the participants were standing on one leg. The COP’s location was determined using an elliptical approximation of the foot region, with the X- and Y-axes serving as the minor and major axes, respectively. **Supplementary Material A** depicts the specific data estimation methods used in this study. The angles of each joint of the body were estimated from full-body images using OpenPose (28). The angles were estimated for 10 joints of each participant. **Figure 3** depicts the estimated positions and names of the joints.

### 2.5 Classification using the Support Vector Machine

From the measured data, 16 variables were extracted; they are listed in **Table 2**. These variables include the movements and complexity of COP sway (*Len_total path_*, *M_cop speed_*, *COP_entropy_*, and *COP_convex hull_*), total balance ability (*Len_max trial_*), difference between body movement and COP variation (*COM* - *COP*), and relationship between body parts and COP movements (*Corr_neck_*, *Corr_shoulder_*, *Corr_right elbow_*, *Corr_left elbow_*, *Corr_right trunk_*, *Corr_left trunk_*, *Corr_right hip_*, *Corr_left hip_*, *Corr_right knee_*, and *Corr_left knee_*). **Supplementary Material B** clarifies the reasons for selecting each explanatory variable and calculation method.

**Table 2:** Explanatory variables for SVM.

Category	Variables	Explanation
**COP sway**	*Len_totalpath_*	The total length of COP sway path
	*M_cop speed_*	Mean speed in COP sway
	*COP_entropy_*	Approximate entropy of COP sway
	*COP_convex hull_*	Convex hull of COP sway
**Overall balance**	*Len_max trial_*	Total time of longest one-leg standing time during trials
	*COM - COP*	The distance between Center of Mass (COM) of the body and COP
**Correlation between COP and joint angles**	*Corr_neck_*	Correlation between change in COP and change in neck angle to the horizontal plane
	*Corr_shoulder_*	Correlation between change in COP and change in shoulder angle to the horizontal plane
	*Corr_right elbow_*	Correlation between change in COP and change in right elbow joint angle
	*Corr_left elbow_*	Correlation between change in COP and change in left elbow joint angle
	*Corr_right trunk_*	Correlation between change in COP and change in angle between the midline of trunk and the right hip joint
	*Corr_left trunk_*	Correlation between change in COP and change in angle between the midline of trunk and the left hip joint
	*Corr_right hip_*	Correlation between change in COP and change in right hip joint angle
	*Corr_left hip_*	Correlation between change in COP and change in left hip joint angle
	*Corr_right knee_*	Correlation between change in COP and change in right knee joint angle
	*Corr_left knee_*	Correlation between change in COP and change in left knee joint angle

SVM, Support Vector Machine, COP, center of pressure.

We examined the process of transitioning from standing on two legs to standing on one leg, since we consider it a feed-forward process that is necessary to achieve the one-legged standing outcome. Therefore, the inherent diversity of the transitioning process was noted by everyone. We computed these variables (excluding *Len_max trial_*) for the initial two seconds of each participant’s first trial.

Support Vector Machine (SVM) is a popular machine learning method used in many classification problems (29). We used 16 explanatory variables to perform classification learning using SVM to determine whether participants belonged to the High autistic trait group or Low autistic trait group. Subsequently, we simultaneously selected the 16 explanatory variables and optimized the hyperparameter to screen High autistic trait group and, thereby, improve the performance and generalization of all SVM classification models.

Further, classification models were built for the following three sets of explanatory variables:

1. Conventional variables: COP sway + overall balance (variables based on earlier studies (30–36).

2. Proposed variables: Correlation between COP and joint angles (variables added by the current study).

3. Combined variables: All the explanatory variables.

The probabilities of participants being classified into the High autistic trait group were calculated using an SVM model. The performance of the classification models was evaluated using leave-one-out cross-validation (LOOCV). **Supplementary Material C** presents more information on the construction and evaluation of these models.

### 2.6 Statistical analyses

Statistical analyses were performed using the SPSS software, version 27.0 (IBM, Armonk, NY). Accordingly, descriptive statistics were calculated for all samples. The differences in age and SRS-2 scores between the two groups were analyzed using an independent sample t-test. Further, the difference in sex ratio was analyzed using the χ2 test. We calculated the accuracy, sensitivity, and specificity of models based on LOOCV classification using the best classification model. Further, we used Shapley additive explanations (SHAP) to identify the important features of each set of explanatory variable categories (i.e., conventional, proposed, and combined variables). This indicated the impact of each feature on the classifier’s output and the range of each variable to increase the probability of participants being classified into the High autistic trait group or Low autistic trait group. Subsequently, we calculated Spearman’s rank correlation coefficients using the total SRS score and the participants’ probabilities of being classified into the High autistic trait group. For all statistical tests, a significance threshold of *p* <.05 was adopted.

## 3 Results

**Table 3** presents the results of the best classification model for each set of explanatory variable categories. The accuracy, sensitivity, and specificity scores of all sets of all variables, except the accuracy and sensitivity scores of conventional variables, are 1.000.

**Table 3:** The best results of LOOCV.

	Accuracy	Sensitivity	Specificity	Selected Variables
**Conventional variables** (0.001)	0.976	0.842	1.000	High autistic trait group ~ *Len_max trial_* + *COP_convex hull_* + *COM - COP*
**Proposed variables** (0.01)	1.000	1.000	1.000	High autistic trait group ~ *Corr_shoulder_* + *Corr_right trunk_* + *Corr_right knee_*
**Combined categories** (0.01)	1.000	1.000	1.000	High autistic trait group ~ *COP_convex hull_* + *COM - COP* + *Corr_shoulder_* + *Corr_right hip_* + *Corr_right knee_*

LOOCV, leave-one-out cross-validation.

High autistic trait group: Result of classifying whether the participant had high autistic trait.

Conventional variables: a set of the explanatory variables in “COP sway” and “Overall balance” in **Table 2**.

Proposed variables: a set of the explanatory variables in “Correlation between COP and joint angles” in **Table 2**.

Combined variables: Set of explanatory variables for all categories in **Table 2**.

The numbers below the set of variables represent the hyperparameters of an SVM with a linear kernel. The value of is a Cost parameter.

See **Table 2** for the variable names.

**Figure 4:**
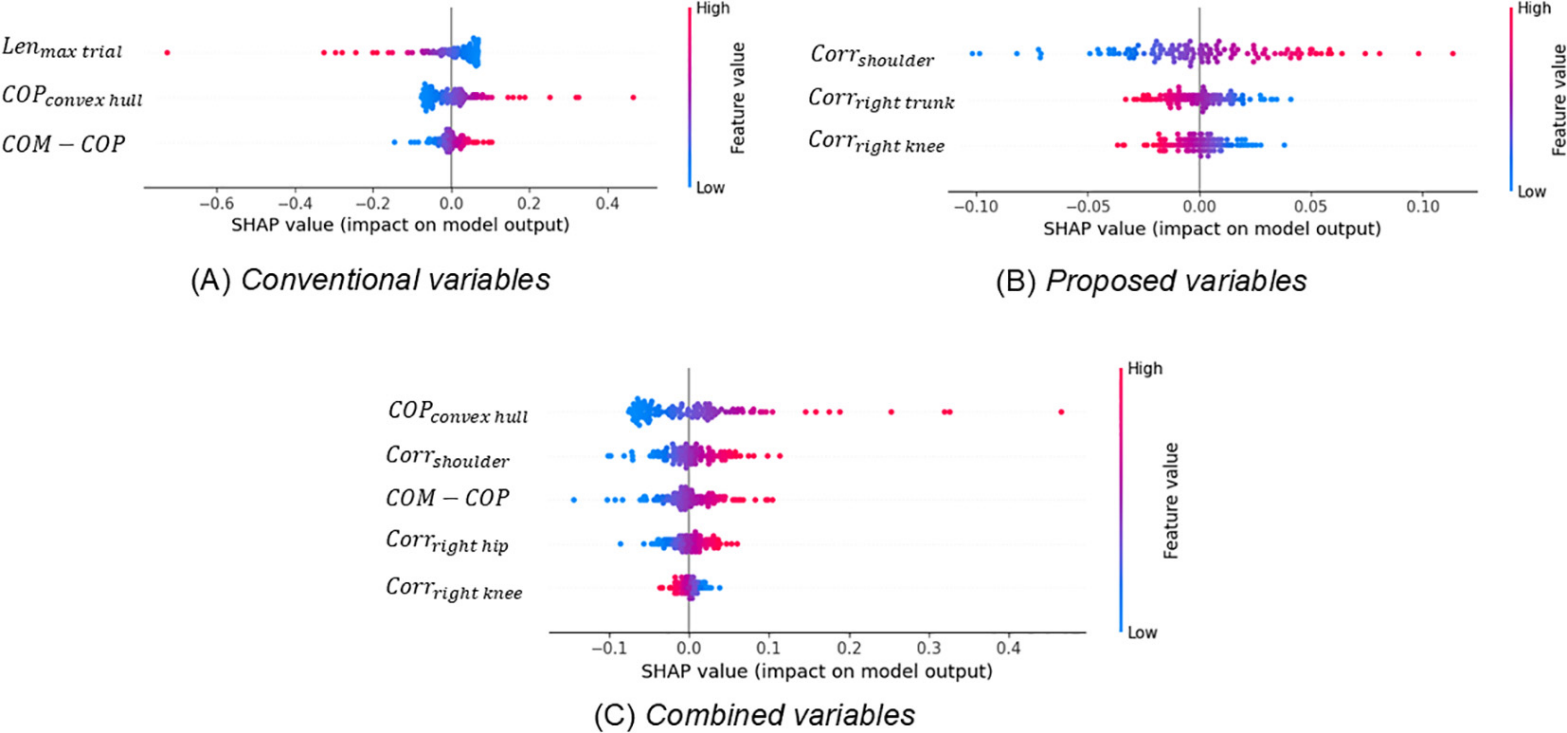
Each feature’s impact on the models quantified using Shapley additive explanation values.

**Figure 4** depicts the SHAP values of the selected variables of each SVM model and their impacts for all participants for three sets of explanatory variable categories. Among the 16 variables, 3–5 were selected from each SVM model. The classification model with conventional variables included variables such as the duration of the longest one-leg stand in trials (*Len_max trial_*), area of COP sway (*COP_convex hull_*), and association between body movements and COP variations (*COM - COP*). The classification model with proposed variables included variables indicating the correlation between the shoulder angle and COP (*Corr_shoulder_*), correlation between the right trunk angle and COP (*Corr_right trunk_*), and correlation between the right knee angle and COP (*Corr_right knee_*). Finally, the classification model with combined variables included the variables for the area of COP sway (*COP_convex hull_*), association between body movement and COP variation (*COM - COP*), correlation between the shoulder angle and COP (*Corr_shoulder_*), correlation between the right hip angle and COP (*Corr_right hip_*), and correlation between the right knee angle and COP (*Corr_right knee_*). Even when the same variable is selected from different sets of explanatory variable categories, its contribution to the classification differs across variable sets. Among conventional variables and combined variables, the *COP_convex hull_* (which is related to the area of COP sway) and *COM - COP* (which is related to the association between body movement and COP variation) are commonly selected. Among conventional variables, *Len_max trial_* is selected as the more important variable compared to the two variables *COP_convex hull_* and *COM - COP*. However, among combined variables, *COP_convex hull_* is selected as the most important variable (**Figures 4A, C**). Further, among proposed variables and combined variables, the shoulder joint and right knee are commonly selected as the most important variables. Among proposed variables, both the smaller values of *Corr_shoulder_* and *Corr_right knee_* contributed to the categorization of the High autistic trait group. However, among combined variables, a larger value of *Corr_shoulder_* contributed to High autistic trait group categorization (**Figures 4B, C**).

**Figure 5:**
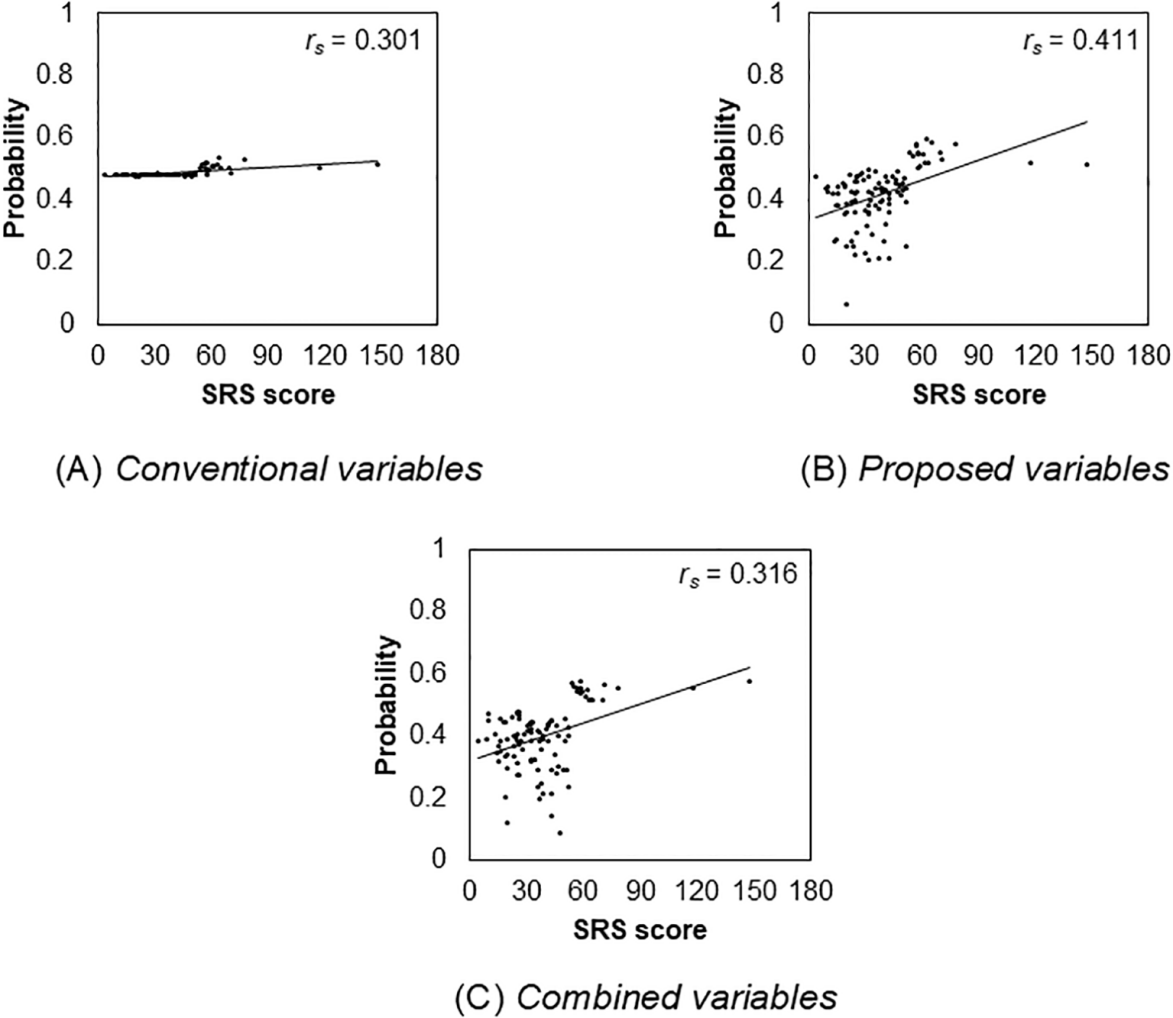
Relationship between the total Social Responsiveness Scale score and the probability of autism spectrum disorder in each variable.

The total SRS score was positively correlated with probability of High autistic trait group in each set of explanatory variable categories (conventional: *r_s_* (124) = 0.301, *p* = 0.002; proposed: *r_s_* (124) = 0.411, *p* < 0.001; combined: *r_s_* (124) = 0.316, *p* < 0.001). Further, the probabilities of high autistic trait group in the proposed variables category are relatively highly correlated with the total SRS score. However, the correlation between conventional variables and the total SRS score was low. **Figure 5** depicts these distributions. Finally, the distribution of the conventional variables was very different from that of the other two variables.

## 4 Discussion

This study assessed the potential usefulness of machine learning in evaluating movements during a one-legged standing test conducted for predicting high autistic trait group. The LOOCV results computed using the best classification model indicated the high accuracy, sensitivity, and specificity of variables. We identified the shoulder, hip, and trunk to be important variables in explaining the balance status of children with high autistic trait. The probabilities of High autistic trait group, which were calculated using each set of explanatory variable categories (i.e., conventional, proposed, and combined variables) and the SVM model, were correlated with the autistic traits assessed by SRS. It is noted that the screening was completed in a very short time. These results demonstrate the potential of using the one-legged standing test and machine learning as autism screening techniques.

Eye-tracking is one of the most frequently applied machine learning technique in ASD screening. According to Wei et al. (37), machine learning classification using eye tracking has an accuracy of 81%, a specificity of 79%, and a sensitivity of 84% in distinguishing individuals belonging to ASD and TD groups. However, the proposed and combined variables in this study had 100% accuracy, specificity, and sensitivity. Since screening rarely achieves 100% accuracy, sensitivity, and specificity, the difficulty of the one-legged standing test is the core aspect of ASD. These data support the use of this test in ASD screening. Moreover, this study’s results are relevant in practice, since its participants were 5-year-old children and the task took only a short time to complete.

Traditional assessments of the balance ability of children with high autistic trait commonly involve examinations of the entire body, from the lower to upper body (38). However, this study revealed that, compared to other parts of the body, the shoulder joints, hips, and waist contributed more to the body’s balance ability. Although it is important to examine the entire body, the results suggest that paying specific attention to the feed-forward strategy to control the center of gravity using the shoulder and waist at the beginning of the one-legged standing test contributes to the efficient screening of children with high autistic trait. Further, in screening using the one-legged standing test, focusing on shoulder and waist movements can facilitate the efficient screening of children with high autistic trait.

The SRS was rated according to a subjective evaluation by mothers in this study. However, the assessment performed in this study (i.e., standing on one leg) was based on an objective evaluation. Since the methods for assessing SRS and standing on one leg are completely different, assessing both is potentially more useful for predicting children with high autistic trait.

This study has several limitations, and these should be addressed by future research. First, the sample size was relatively small; accordingly, the results may not be sufficiently comprehensive. Future studies on larger sample sizes can provide more meaningful data on the potential use of drawing skills in screening of children with high autistic trait. Second, we did not collect any information regarding participants’ intelligence quotient (IQ). However, all the participants attended mainstream preschools without any intellectual impairment. In addition, motor impairments were found in both children with ASD having comorbid intellectual disability and those with ASD having average or above-average IQ (39). Accordingly, the observed motor difficulties commonly co-occur with a diagnosis of ASD, are ASD-specific features, and are not entirely due to impaired intellectual functioning. Third, the SRS is clearly just a screening tool for ASD. In fact, a substantial proportion of children with SRS scores lower than the cut-off of 55 (about 20%) have ASD and vice versa a considerable proportion of children with SRS scores higher than 55 (about 20%) do not have ASD (26). While our model provides a useful screening tool, it should not be used in isolation for diagnosis. Instead, it should be considered as part of a comprehensive assessment process that includes clinical evaluation and other standardized measures. Based on the results of this study, we intend to consider developing a continuous SRS score prediction model as a next step. Moreover, integrating more robust diagnostic tools for ASD, such as clinical assessments or standardized behavioral tests, alongside the SRS-2, would improve the reliability of the screening process. There are also limitations due to the sample size of the above-threshold data (i.e., data of the High autistic trait group). With only around 20 above-threshold samples and highly imbalanced sample overall, we were forced to use LOOCV to avoid polarization of prediction toward the Low autistic trait group. Although LOOCV is suitable for small datasets, it has drawbacks. It is computationally expensive and time consuming, especially for large datasets or complex models. LOOCV can also produce optimistic performance estimates due to the small test set in each iteration. In addition, it can be unstable when the dataset contains noise or outliers, as individual data points can disproportionately influence the results. These limitations emphasize the need for cautious interpretation of our results. Future research using more balanced dataset with larger samples above the threshold might require model revisions and could potentially yield more robust and generalizable results. While the study focuses on the support vector machine (SVM), comparing its performance with other algorithms, such as random forests or deep learning techniques, could identify the most effective approach for this type of screening. Furthermore, validation using external datasets from different regions or institutions would help confirm whether the findings hold across different populations.

In conclusion, this study revealed the effectiveness of using machine learning to evaluate the balance ability of 5-year-old children standing on one leg as a means of predicting high autistic trait. In addition, we emphasize the significance of focusing specifically on the shoulder and waist movements during the one-legged standing test to facilitate the efficient predicting of children with high autistic trait. One of the greatest advantages that research in this field can provide is the potential of identifying additional biomarkers as potential predictors of ASD and ensuring new prospective for early diagnosis (40). Further studies incorporating a broader range of balance cues are necessary to comprehensively determine the potential usefulness of considering balance ability in screening procedures of children with high autistic trait.

## Data Availability

The original contributions presented in the study are included in the article/**Supplementary Material**. Further inquiries can be directed to the corresponding author.

## References

[1] American Psychiatric Association (APA). Diagnostic and Statistical Manual of Mental Disorders. 5th. Arlington, VA: American Psychiatric Publishing (2013) p. 5–25.

[2] Maenner MJ Warren Z Williams AR Amoakohene E Bakian AV Bilder DA. Prevalence and characteristics of autism spectrum disorder among children aged 8 years — Autism and developmental disabilities monitoring network, 11 sites, United States, 2020. MMWR Surveill Summ. (2023) 72:1–14. doi: 10.15585/mmwr.ss7202a1 PMC10042614 36952288

[3] Cakir J Frye RE Walker SJ. The lifetime social cost of autism: 1990–2029. Res Autism Spec Disord. (2020) 72:101502. doi: 10.1016/j.rasd.2019.101502

[4] Reichow B. Overview of meta-analyses on early intensive behavioral intervention for young children with autism spectrum disorders. J Autism Dev Disord. (2012) 42:512–20. doi: 10.1007/s10803-011-1218-9 21404083

[5] Rogers SJ Estes A Lord C Munson J Rocha M Winter J. A multisite randomized controlled two-phase trial of the early start Denver Model compared to treatment as usual. J Am Acad Child Adolesc Psychiatry. (2019) 58:853–65. doi: 10.1016/j.jaac.2019.01.004 PMC12088912 30768394

[6] Smith T Iadarola S. Evidence base update for autism spectrum disorder. J Clin Child Adolesc Psychol. (2015) 44:897–922. doi: 10.1080/15374416.2015.1077448 26430947

[7] Warren Z McPheeters ML Sathe N Foss-Feig JH Glasser A Veenstra-VanderWeele J. A systematic review of early intensive intervention for autism spectrum disorders. Pediatrics. (2011) 127:e1303–11. doi: 10.1542/peds.2011-0426 21464190

[8] Landa RJ Gross AL Stuart EA Faherty A. Developmental trajectories in children with and without autism spectrum disorders: The first 3 years. Child Dev. (2013) 84:429–42. doi: 10.1111/j.1467-8624.2012.01870.x PMC4105265 23110514

[9] Ozonoff S Young GS Landa RJ Brian J Bryson S Charman T. Diagnostic stability in young children at risk for autism spectrum disorder: A baby siblings research consortium study. J Child Psychol Psychiatry. (2015) 56:988–98. doi: 10.1111/jcpp.12421 PMC4532646 25921776

[10] Maenner MJ Shaw KA Baio J Washington A Patrick M DiRienzo M. Prevalence of autism spectrum disorder among children aged 8 years — Autism and developmental disabilities monitoring network, 11 sites, United States, 2016. MMWR CDC Surveill Summ. (2020) 69:1–12. doi: 10.15585/mmwr.ss6904a1 PMC7119644 32214087

[11] Paillard T. Plasticity of the postural function to sport and/or motor experience. Neurosci Biobehav Rev. (2017) 72:129–52. doi: 10.1016/j.neubiorev.2016.11.015 27894829

[12] Divandari N Bird ML Vakili M Jaberzadeh S. The association between dynamic balance and executive function: Which dynamic balance test has the strongest association with executive function? A systematic review and meta-analysis. Curr Neurol Neurosci Rep. (2024) 24:151–61. doi: 10.1007/s11910-024-01340-3 PMC11143012 38730213

[13] Horak FB. Postural orientation and equilibrium: What do we need to know about neural control of balance to prevent falls? Age Ageing. (2006) 35 Supplement 2:ii7–ii11. doi: 10.1093/ageing/afl077 16926210

[14] Pijnappels M Reeves ND Maganaris CN van Dieën JH. Tripping without falling; lower limb strength, a limitation for balance recovery and a target for training in the elderly. J Electromyogr Kinesiol. (2008) 18:188–96. doi: 10.1016/j.jelekin.2007.06.004 17761436

[15] Whittingham K Fahey M Rawicki B Boyd R. The relationship between motor abilities and early social development in a preschool cohort of children with cerebral palsy. Res Dev Disabil. (2010) 31:1346–51. doi: 10.1016/j.ridd.2010.07.006 20674264

[16] Fournier KA Hass CJ Naik SK Lodha N Cauraugh JH. Motor coordination in autism spectrum disorders: A synthesis and meta-analysis. J Autism Dev Disord. (2010) 40:1227–40. doi: 10.1007/s10803-010-0981-3 20195737

[17] Kaur M Srinivasan S M Bhat N. Comparing motor performance, praxis, coordination, and interpersonal synchrony between children with and without Autism Spectrum Disorder (ASD). Res Dev Disabil. (2018) 72:79–95. doi: 10.1016/j.ridd.2017.10.025 29121516 PMC5743591

[18] Doumas M McKenna R Murphy B. Postural control deficits in autism spectrum disorder: The role of sensory integration. J Autism Dev Disord. (2016) 46:853–61. doi: 10.1007/s10803-015-2621-4 26446773

[19] Fournier KA Kimberg CI Radonovich KJ Tillman MD Chow JW Lewis MH. Decreased static and dynamic postural control in children with autism spectrum disorders. Gait Posture. (2010) 32:6–9. doi: 10.1016/j.gaitpost.2010.02.007 20400311 PMC2919314

[20] Yoshimura N Muraki S Iidaka T Oka H Horii C Kawaguchi H. Prevalence and co-existence of locomotive syndrome, sarcopenia, and frailty: The third survey of Research on Osteoarthritis/Osteoporosis Against Disability (ROAD) study. J Bone Miner Metab. (2019) 37:1058–66. doi: 10.1007/s00774-019-01012-0 31222550

[21] Araujo CG de Souza E Silva CG Laukkanen JA Fiatarone Singh M Kunutsor SK Myers J. Successful 10-second one-legged stance performance predicts survival in middle-aged and older individuals. Br J Sports Med. (2022) 56:975–80. doi: 10.1136/bjsports-2021-105360 35728834

[22] Khanal P He L Stebbings GK Onambele-Pearson GL Degens H Williams AG. Static one-leg standing balance test as a screening tool for low muscle mass in healthy elderly women. Aging Clin Exp Res. (2021) 33:1831–9. doi: 10.1007/s40520-021-01818-x PMC8249245 33715139

[23] Travers BG Powell PS Klinger LG Klinger MR. Motor difficulties in autism spectrum disorder: Linking symptom severity and postural stability. J Autism Dev Disord. (2013) 43:1568–83. doi: 10.1007/s10803-012-1702-x 23132272

[24] Graham SA Abbott AE Nair A Lincoln AJ Müller R-A Goble DJ. The influence of task difficulty and participant age on balance control in ASD. J Autism Dev Disord. (2015) 45:1419–27. doi: 10.1007/s10803-014-2303-7 25381191

[25] Constantino J Gruber C. The social responsiveness scale. Los Angeles: Western psychol Serv. (2002).

[26] Kamio Y Inada N Moriwaki A Kuroda M Koyama T Tsujii H. Quantitative autistic traits ascertained in a national survey of 22 529 Japanese schoolchildren. Acta Psychiatr Scand. (2013) 128:45–53. doi: 10.1111/acps.12034 23171198 PMC3604131

[27] Shirama A Stickley A Kamio Y Nakai A Takahashi H Saito A. Emotional and behavioral problems in Japanese preschool children with motor coordination difficulties: The role of autistic traits. Eur Child Adolesc Psychiatry. (2022) 31:979–90. doi: 10.1007/s00787-021-01732-7 33566188

[28] Cao Z Hidalgo G Simon T Wei S-E Sheikh Y. OpenPose: Realtime multi-person 2D pose estimation using part affinity fields. IEEE Trans Pattern Anal Mach Intell. (2021) 43:172–86. doi: 10.1109/TPAMI.2019.2929257 31331883

[29] Eman D Emanuel AWR. Machine learning classifiers for autism spectrum disorder: A review 4th International Conference on Information Technology. Inf Syst Electrical Eng (ICITISEE). (2019), 255–60. doi: 10.1109/ICITISEE48480.2019.9003807

[30] Smoot Reinert S Jackson K Bigelow K. Using posturography to examine the immediate effects of vestibular therapy for children with autism spectrum disorders: A feasibility study. Phys Occup Ther Pediatr. (2015) 35:365–80. doi: 10.3109/01942638.2014.975313 25374155

[31] Morris SL Foster CJ Parsons R Falkmer M Falkmer T Rosalie SM. Differences in the use of vision and proprioception for postural control in autism spectrum disorder. Neuroscience. (2015) 307:273–80. doi: 10.1016/j.neuroscience.2015.08.040 26314635

[32] Sun R Hsieh KL Sosnoff JJ. Fall risk prediction in multiple sclerosis using postural sway measures: A machine learning approach. Sci Rep. (2019) 9:16154. doi: 10.1038/s41598-019-52697-2 31695127 PMC6834625

[33] Winter DA. Human balance and posture control during standing and walking. Gait Posture. (1995) 3:193–214. doi: 10.1016/0966-6362(96)82849-9

[34] Hsue B-J Miller F Su F-C. The dynamic balance of the children with cerebral palsy and typical developing during gait. Part I: Spatial relationship between COM and COP trajectories. Gait Posture. (2009) 29:465–70. doi: 10.1016/j.gaitpost.2008.11.007 19111469

[35] Montesinos L Castaldo R Pecchia L. On the use of approximate entropy and sample entropy with centre of pressure time-series. J Neuroeng Rehabil. (2018) 15:116. doi: 10.1186/s12984-018-0465-9 30541587 PMC6291990

[36] Molloy CA Dietrich KN Bhattacharya A. Postural stability in children with autism spectrum disorder. J Autism Dev Disord. (2003) 33:643–52. doi: 10.1023/b:jadd.0000006001.00667.4c 14714933

[37] Wei Q Cao H Shi Y Xu X Li T. Machine learning based on eye-tracking data to identify autism Spectrum Disorder: A systematic review and meta-analysis. J BioMed Inform. (2023) 137:104254. doi: 10.1016/j.jbi.2022.104254 36509416

[38] Baeza-Velasco C Cohen D Hamonet C Vlamynck E Diaz L Cravero C. Autism, joint hypermobility-related disorders and pain. Front Psychiatry. (2018) 9:656. doi: 10.3389/fpsyt.2018.00656 30581396 PMC6292952

[39] Green D Baird G Barnett AL Henderson L Huber J Henderson SE. The severity and nature of motor impairment in Asperger’s syndrome: A comparison with Specific developmental disorder of Motor Function. J Child Psychol Psychiatry. (2002) 43:655–68. doi: 10.1111/1469-7610.00054 12120861

[40] Simeoli R Rega A Cerasuolo M Nappo R Marocco D. Using machine learning for motion analysis to early detect autism spectrum disorder: A systematic review. Rev J Autism Dev Disord. (2024). doi: 10.1007/s40489-024-00435-4

