# Supplementary material for: Machine learning’s effectiveness in evaluating movement in one-legged standing test for predicting high autistic trait
Source: Front Psychiatry. 2024 Oct 17;15:1464285. doi: 10.3389/fpsyt.2024.1464285 (PMC11524919; doi:10.3389/fpsyt.2024.1464285)
Supplement: Supplementary file 1 [file DataSheet1.docx]

Supplementary Material

# 1 Supplementary Material A

The procedure for determining the center of pressure (COP) while standing on one leg can be summarized as follows:

Step 1: The area of the grounded foot during the one-legged stand is estimated from the entire dataset.

Step 2: The estimated foot area is tracked throughout the one-legged stand.

Step 3: The interval from the time-series data is extracted during which the participant was standing on one leg.

Step 4: The time-series of COP is estimated within the extracted interval.

Note. If the trial of standing on one leg was conducted on the left foot, the pressure and image data must be inverted from left to right before Step 1.

# 2 Supplementary Material B

Several previous studies have assessed the center of pressure (COP) in. These studies have reported that children with high autistic trait have larger COP sway displacements (Smoot Reinert et al., 2015), sway areas (Mache and Todd, 2016) and sway velocities (Morris et al., 2015) compared to age-matched typically developing children. In this study, we used as explanatory variables those findings that could be useful features when applied to the data obtained in the focused range (initial two seconds in the first trial).

We chose the total length of COP sway path in the focused range (${Len}_{total path}$) and the mean of speed in COP sway ($M_{cop speed}$) as indicators for assessment of the magnitude of postural sway.

We chose the sample entropy of COP sway (${COP}_{entropy}$) and convex hull of COP sway (${COP}_{convex hull}$), as indicators for assessment of the complexity of postural sway. The sample entropy is commonly used to assess complexity in COP time-series in different groups and test conditions (Smoot Reinert et al., 2015; Montesinos et al., 2018; Sun et al., 2019). The convex hull assesses the magnitude and complexity of COP sway (Molloy et al., 2003).

We chose the total time of longest one-leg standing time during trials (${Len}_{max trial}$) and the distance between Center of Mass (COM) of body and Center of Pressure (COP) ($COM-COP$) as indicators of overall balancing ability. The total time of one-leg standing time is a simple indicator for the assessment. $COM-COP$ is one of the indicators that reflects the association between body movement and COP variation (Winter 1995; Hsue et al., 2009).

The relationship between changes in the center of pressure of the sole of the grounded foot and body movements is undeniable. To analyze the intricate body control performed by multiple joints in unison, we introduced variables that indicate the relationship between the angle changes of individual joints and the changes in the center of pressure. In this study, we selected the simple relationship as the variable, which was the correlation between the amount of change in the center of pressure and the amount of angular change at each joint. Ten joints were chosen for analysis: neck, shoulder, right elbow, left elbow, right trunk, left trunk, right hip, left hip, right knee, and left knee. For the neck and shoulders, the angles made with the horizontal plane were used to calculate the correlation. For the other eight joints, the medial angle of each joint estimated from the participant’s frontal images was used to calculate the correlations. The corresponding variables are ${Corr}_{neck}$, ${Corr}_{shoulder}$, ${Corr}_{right elbow}$, ${Corr}_{left elbow}$, ${Corr}_{right trunk}$, ${Corr}_{left trunk}$, ${Corr}_{right hip}$, ${Corr}_{left hip}$, ${Corr}_{right knee}$, and ${Corr}_{left knee}$.

See **Table S1** for the calculation formulae of each explanatory variable.

See **Figure S1** for the algorithm to calculate the ${COP}_{entropy}$.

See **Figure S2** for the algorithm to calculate the ${COP}_{convex hull}$.

# 3 Supplementary Material C

## Classification model training to predicting high autistic trait group

The model for classifying whether participants were suspected of having high autistic trait was trained using an SVM algorithm with the 16 explanatory variables (Table 2 and Table S1). We used the ksvm function from the Kernlab library in R version 4.2.3 to run the SVM, using the linear kernel. In the linear kernel, there is one hyperparameter (cost). A grid search was performed to determine the optimal hyperparameter. SVM parameter used linear kernel with cost (C) of {0.0001, 0.001, 0.01, 0.1, 1, 10}. To reduce computational time, variable selection was performed using the backward-forward selection method for each hyperparameter combination. For variable selection, leave-one-out cross-validation errors were compared, and the variables with the smallest cross-validation errors were chosen.

## Performance evaluation of classification models

After the training, the performance of the final classification model was evaluated. To evaluate the performance of the hyperparameter and selected variable sets, leave-one-out cross-validation (LOOCV) was performed on the hyperparameter and variable sets.

During the LOOCV process, the data for each participant were sequentially removed from the dataset. The SVM classification model was trained using the remaining data. Subsequently, the performance of the model was tested using data that were initially removed.

Smoot Reinert, S., Jackson, K., & Bigelow, K. (2015). Using posturography to examine the immediate effects of vestibular therapy for children with autism spectrum disorders: a feasibility study. Physical & occupational therapy in pediatrics, 35(4), 365-380. <https://doi.org/10.3109/01942638.2014.975313>

Mache, M. A., & Todd, T. A. (2016). Gross motor skills are related to postural stability and age in children with autism spectrum disorder. Research in Autism Spectrum Disorders, 23, 179-187. <https://doi.org/10.1016/j.rasd.2016.01.001>

Morris, S. L., Foster, C. J., Parsons, R., Falkmer, M., Falkmer, T., & Rosalie, S. M. (2015). Differences in the use of vision and proprioception for postural control in autism spectrum disorder. Neuroscience, 307, 273-280. <https://doi.org/10.1016/j.neuroscience.2015.08.040>

Montesinos, L., Castaldo, R., & Pecchia, L. (2018). On the use of approximate entropy and sample entropy with centre of pressure time-series. Journal of neuroengineering and rehabilitation, 15, 1-15. <https://doi.org/10.1186/s12984-018-0465-9>

Sun, R., Hsieh, K. L., & Sosnoff, J. J. (2019). Fall risk prediction in multiple sclerosis using postural sway measures: a machine learning approach. Scientific reports, 9(1), 16154. <https://doi.org/10.1038/s41598-019-52697-2>

Molloy, C. A., Dietrich, K. N., & Bhattacharya, A. (2003). Postural stability in children with autism spectrum disorder. Journal of autism and developmental disorders, 33, 643-652. [https://doi.org/10.1023/B:JADD.0000006001.00667.4c](https://doi.org/10.1023/b:jadd.0000006001.00667.4c)

Winter, D. A. (1995). Human balance and posture control during standing and walking. Gait & posture, 3(4), 193-214. <https://doi.org/10.1016/0966-6362(96)82849-9>

Hsue, B. J., Miller, F., & Su, F. C. (2009). The dynamic balance of the children with cerebral palsy and typical developing during gait. Part I: Spatial relationship between COM and COP trajectories. Gait & posture, 29(3), 465-470. <https://doi.org/10.1016/j.gaitpost.2008.11.007>

**Table S1*.* Formulae for explanatory variables for SVM**

| Category | Variables | Formula |
| --- | --- | --- |
| COP sway | ${Len}_{total path}$ | $\sum_{i=1}^{n-1} {CS}_{i}$,  ${CS}_{i}=\sqrt{\left( h_{i+1}-h_{i} \right)^{2}+\left( v_{i+1}-v_{i} \right)^{2}} \left( 1\leq i\leq n-1 \right)$,  where $h_{i} is a horizontal position of COP (0\leq h_{i}\leq1)$, $v_{i} is a vertical position of COP (0\leq v_{i}\leq1)$ |
|  | $M_{cop speed}$ | $\frac{1}{n-1}\sum_{i=1}^{n-1} {CS}_{i}$,  ${CS}_{i}=\frac{\sqrt{\left( h_{i+1}-h_{i} \right)^{2}+\left( v_{i+1}-v_{i} \right)^{2}}}{unit time} \left( 1\leq i\leq n-1 \right)$,  where $h_{i} is a horizontal position of COP (0\leq h_{i}\leq1)$, $v_{i} is a vertical position of COP (0\leq v_{i}\leq1)$ |
|  | ${COP}_{entropy}$ | $ApEn\left( m, r, D \right) \left( see Figure S1 \right),$  $where m is a sequence length \left( m= 2 \right)$,  $r is a tolerance \left( r= 0.2*standard deviation of D \right),$  $D is time-series data of COP.$ |
|  | ${COP}_{convex hull}$ | *ConvexHullArea*$\left( Points \right) \left( see Figure S2 \right),$  $where Points is a list of 2D points of COP.$ |
| Overall balance | ${Len}_{max trial}$ | $\max_{1\leq i\leq4} ({LT}_{i})$,  where ${LT}_{i} is a time of one leg standing in a trial i$  $(2\leq{PT}_{i}\leq20)$ |
|  | $COM-COP$ | $\sum_{i=1}^{n-1} abs({COM}_{i}-{COP}_{i})$,  ${COM}_{i}=h_{i+1}-h_{i}, {COP}_{i}=v_{i+1}-v_{i} \left( 1\leq i\leq n-1 \right)$,  where $h_{i} is a horizontal position of COM (0\leq h_{i}\leq1)$, $v_{i} is a horizontal position of COP (0\leq v_{i}\leq1)$ |

| Category | Variables | Formula |
| --- | --- | --- |
| Correlation between COP and joint angles | ${Corr}_{neck}$ | $\frac{\frac{1}{n}\sum_{i=1}^{n} {(a}_{i}-\frac{\sum_{k=1}^{n} a_{k}}{n}){(b}_{i}-\frac{\sum_{k=1}^{n} b_{k}}{n})}{\sqrt{\frac{1}{n}\sum_{i=1}^{n} {{(a}_{i}-\frac{\sum_{k=1}^{n} a_{k}}{n})}^{2}}\sqrt{\frac{1}{n}\sum_{i=1}^{n} {{(b}_{i}-\frac{\sum_{k=1}^{n} b_{k}}{n})}^{2}}}$,  where $a_{i} and a_{k} are movements of COP \left( 0\leq a_{i}, a_{k} \right),$  $b_{i} , b_{k} are change in neck angle to the horizontal plane$  $(-90^{\circ}\leq b_{i}, b_{k}\leq90^{\circ})$ |
|  | ${Corr}_{shoulder}$ | $\frac{\frac{1}{n}\sum_{i=1}^{n} {(a}_{i}-\frac{\sum_{k=1}^{n} a_{k}}{n}){(b}_{i}-\frac{\sum_{k=1}^{n} b_{k}}{n})}{\sqrt{\frac{1}{n}\sum_{i=1}^{n} {{(a}_{i}-\frac{\sum_{k=1}^{n} a_{k}}{n})}^{2}}\sqrt{\frac{1}{n}\sum_{i=1}^{n} {{(b}_{i}-\frac{\sum_{k=1}^{n} b_{k}}{n})}^{2}}}$,  where $a_{i} and a_{k} are movements of COP \left( 0\leq a_{i}, a_{k} \right),$  $b_{i} , b_{k} are change in shoulder angle to the horizontal plane$  $(-90^{\circ}\leq b_{i}, b_{k}\leq90^{\circ})$ |
|  | ${Corr}_{right elbow}$ | $\frac{\frac{1}{n}\sum_{i=1}^{n} {(a}_{i}-\frac{\sum_{k=1}^{n} a_{k}}{n}){(b}_{i}-\frac{\sum_{k=1}^{n} b_{k}}{n})}{\sqrt{\frac{1}{n}\sum_{i=1}^{n} {{(a}_{i}-\frac{\sum_{k=1}^{n} a_{k}}{n})}^{2}}\sqrt{\frac{1}{n}\sum_{i=1}^{n} {{(b}_{i}-\frac{\sum_{k=1}^{n} b_{k}}{n})}^{2}}}$,  where $a_{i} and a_{k} are movements of COP \left( 0\leq a_{i}, a_{k} \right),$  $b_{i} , b_{k} are change in right elbow joint angle$  $(0^{\circ}\leq b_{i}, b_{k}\leq180^{\circ})$ |
|  | ${Corr}_{left elbow}$ | $\frac{\frac{1}{n}\sum_{i=1}^{n} {(a}_{i}-\frac{\sum_{k=1}^{n} a_{k}}{n}){(b}_{i}-\frac{\sum_{k=1}^{n} b_{k}}{n})}{\sqrt{\frac{1}{n}\sum_{i=1}^{n} {{(a}_{i}-\frac{\sum_{k=1}^{n} a_{k}}{n})}^{2}}\sqrt{\frac{1}{n}\sum_{i=1}^{n} {{(b}_{i}-\frac{\sum_{k=1}^{n} b_{k}}{n})}^{2}}}$,  where $a_{i} and a_{k} are movements of COP \left( 0\leq a_{i}, a_{k} \right),$  $b_{i} , b_{k} are change in left elbow joint angle$  $(0^{\circ}\leq b_{i}, b_{k}\leq180^{\circ})$ |
|  | ${Corr}_{right trunk}$ | $\frac{\frac{1}{n}\sum_{i=1}^{n} {(a}_{i}-\frac{\sum_{k=1}^{n} a_{k}}{n}){(b}_{i}-\frac{\sum_{k=1}^{n} b_{k}}{n})}{\sqrt{\frac{1}{n}\sum_{i=1}^{n} {{(a}_{i}-\frac{\sum_{k=1}^{n} a_{k}}{n})}^{2}}\sqrt{\frac{1}{n}\sum_{i=1}^{n} {{(b}_{i}-\frac{\sum_{k=1}^{n} b_{k}}{n})}^{2}}}$,  where $a_{i} and a_{k} are movements of COP \left( 0\leq a_{i}, a_{k} \right),$  $b_{i} , b_{k} are change in angle between the midline of trunk$  $and the right hip joint$  $(0^{\circ}\leq b_{i}, b_{k}\leq180^{\circ})$ |
|  | ${Corr}_{left trunk}$ | $\frac{\frac{1}{n}\sum_{i=1}^{n} {(a}_{i}-\frac{\sum_{k=1}^{n} a_{k}}{n}){(b}_{i}-\frac{\sum_{k=1}^{n} b_{k}}{n})}{\sqrt{\frac{1}{n}\sum_{i=1}^{n} {{(a}_{i}-\frac{\sum_{k=1}^{n} a_{k}}{n})}^{2}}\sqrt{\frac{1}{n}\sum_{i=1}^{n} {{(b}_{i}-\frac{\sum_{k=1}^{n} b_{k}}{n})}^{2}}}$,  where $a_{i} and a_{k} are movements of COP \left( 0\leq a_{i}, a_{k} \right),$  $b_{i} , b_{k} are change in angle between the midline of trunk$  $and the left hip joint$  $(0^{\circ}\leq b_{i}, b_{k}\leq180^{\circ})$ |
|  | ${Corr}_{right hip}$ | $\frac{\frac{1}{n}\sum_{i=1}^{n} {(a}_{i}-\frac{\sum_{k=1}^{n} a_{k}}{n}){(b}_{i}-\frac{\sum_{k=1}^{n} b_{k}}{n})}{\sqrt{\frac{1}{n}\sum_{i=1}^{n} {{(a}_{i}-\frac{\sum_{k=1}^{n} a_{k}}{n})}^{2}}\sqrt{\frac{1}{n}\sum_{i=1}^{n} {{(b}_{i}-\frac{\sum_{k=1}^{n} b_{k}}{n})}^{2}}}$,  where $a_{i} and a_{k} are movements of COP \left( 0\leq a_{i}, a_{k} \right),$  $b_{i} , b_{k} are change in right hip joint angle$  $(0^{\circ}\leq b_{i}, b_{k}\leq180^{\circ})$ |
|  | ${Corr}_{left hip}$ | $\frac{\frac{1}{n}\sum_{i=1}^{n} {(a}_{i}-\frac{\sum_{k=1}^{n} a_{k}}{n}){(b}_{i}-\frac{\sum_{k=1}^{n} b_{k}}{n})}{\sqrt{\frac{1}{n}\sum_{i=1}^{n} {{(a}_{i}-\frac{\sum_{k=1}^{n} a_{k}}{n})}^{2}}\sqrt{\frac{1}{n}\sum_{i=1}^{n} {{(b}_{i}-\frac{\sum_{k=1}^{n} b_{k}}{n})}^{2}}}$,  where $a_{i} and a_{k} are movements of COP \left( 0\leq a_{i}, a_{k} \right),$  $b_{i} , b_{k} are change in left hip joint angle$  $(0^{\circ}\leq b_{i}, b_{k}\leq180^{\circ})$ |
|  | ${Corr}_{right knee}$ | $\frac{\frac{1}{n}\sum_{i=1}^{n} {(a}_{i}-\frac{\sum_{k=1}^{n} a_{k}}{n}){(b}_{i}-\frac{\sum_{k=1}^{n} b_{k}}{n})}{\sqrt{\frac{1}{n}\sum_{i=1}^{n} {{(a}_{i}-\frac{\sum_{k=1}^{n} a_{k}}{n})}^{2}}\sqrt{\frac{1}{n}\sum_{i=1}^{n} {{(b}_{i}-\frac{\sum_{k=1}^{n} b_{k}}{n})}^{2}}}$,  where $a_{i} and a_{k} are movements of COP \left( 0\leq a_{i}, a_{k} \right),$  $b_{i} , b_{k} are change in right knee joint angle$  $(0^{\circ}\leq b_{i}, b_{k}\leq180^{\circ})$ |
|  | ${Corr}_{left knee}$ | $\frac{\frac{1}{n}\sum_{i=1}^{n} {(a}_{i}-\frac{\sum_{k=1}^{n} a_{k}}{n}){(b}_{i}-\frac{\sum_{k=1}^{n} b_{k}}{n})}{\sqrt{\frac{1}{n}\sum_{i=1}^{n} {{(a}_{i}-\frac{\sum_{k=1}^{n} a_{k}}{n})}^{2}}\sqrt{\frac{1}{n}\sum_{i=1}^{n} {{(b}_{i}-\frac{\sum_{k=1}^{n} b_{k}}{n})}^{2}}}$,  where $a_{i} and a_{k} are movements of COP \left( 0\leq a_{i}, a_{k} \right),$  $b_{i} , b_{k} are change in left knee joint angle$  $(0^{\circ}\leq b_{i}, b_{k}\leq180^{\circ})$ |

Notes. SVM = support vector machine. COM = center of mass, we used the coordinates of the center of the human torso as an approximation. COP = center of pressure. *n* = total number of data points in initial two seconds for each participant. $unit time=$the duration between the point $COP_{t}$ and point ${COP}_{t+1}$.

| Algorithm 1: Approximate Entropy (ApEn) |
| --- |
| Inputs:  *D*: Array of length *N* representing the time series data  *m*: Embedding dimension  *r*: Matching tolerance  Output:  The Approximate Entropy of the input data  1: function ApEn(*m, r, D*)  2: *Phi_m* ← Phi(*m, r, D*)  3: *Phi_m_1* ← Phi(m + 1, r, D)  4: *ApEn_value* ← *Phi_m* - *Phi_m_1*  5: return *ApEn_value*  6: end function  7: function Phi(*m, r, D*)  8: *N* ← length(*D*)  9: *phi_sum* ← 0  10: for *i* ← 0 to *N* - *m* do  11: *x_i_* ← *D*[*i* : *i* + *m*]  12: *count* ← 0  13: for *j* ← 0 to *N* - *m* do  14: *x_j_* ← *D*[*j* : *j* + *m*]  15: if max_distance(*x_i_*, *x_j_*) ≤ r then  16: *count* ← *count* + 1  17: end if  18: end for  19: *C_i_* ← *count* / (*N* - *m* + 1)  20: *phi_sum* ← *phi_sum* + log(*C_i_*)  21: end for  22: *Phi_m* ← *phi_sum* / (*N* - *m* + 1)  23: return *Phi_m*  24: end function  25: function max_distance(*vec1, vec2*)  26: *max_dist* ← 0  27: for *k* ← 0 to length(*vec1*) - 1 do  28: *dist* ← \|*vec1*[*k*] - *vec2*[*k*]\|  29: if *dist* > *max_dist* then  30: *max_dist* ← *dist*  31: end if  32: end for  33: return *max_dist*  34: end function |

**Figure S1*.* Pseudocode for approximate entropy calculation.**

| Algorithm 2: Convex Hull Area (ConvexHullArea) |
| --- |
| Inputs:  *Points*: A list of points of 2D coordinates (*x, y*)  Output:  The area of the convex hull  1: function ConvexHullArea(*Points*)  2: *hull* = convex_hull(*Points*)  3: *area* = polygon_area(*hull*)  4: return *area*  5: end function  6: function convex_hull(*Points*)  7: Sort points lexicographically (by *x*, then by *y*)  8: Initialize *lower* to an empty list  9: for each *point* in *Points* do  10: while length(*lower*) ≥ 2 and not ccw(*lower*[-2], *lower*[-1], *point*) do  11: Remove the last *point* from *lower*  12: Append *point* to *lower*  13: Initialize *upper* to an empty list  14: for each *point* in reverse(*Points*) do  15: while length(*upper*) ≥ 2 and not ccw(*upper*[-2], *upper*[-1], *point*) do  16: Remove the last *point* from *upper*  17: Append *point* to *upper*  18: Remove the last *point* from each of *lower* and *upper*  19: return concatenation of *lower* and *upper*  20: end function  21: function ccw(*A, B, C*)  22: return (*C*[1] - *A*[1]) * (*B*[0] - *A*[0]) > (*B*[1] - *A*[1]) * (*C*[0] - *A*[0])  23: end function  24: function polygon_area(*vertices*)  25: *n* = length(*vertices*)  26: *area* = 0  27: for *i* from 0 to *n* - 1 do  28: *x1*, *y1* = *vertices*[*i*][0], *vertices*[*i*][1]  29: *x2*, *y2* = *vertices*[(*i* + 1) % *n*][0], *vertices*[(*i* + 1) % *n*][1]  30: *area* += *x1* * *y2* – *y1* * *x2*  31: return \|*area*\| / 2.0  32: end function |

**Figure S2*.* Pseudocode for convex hull area calculation.**
